# Supplementary material for: Spatial Problem-Solving in Working Dogs: The Combined Effect of Body-Size Awareness, Social Learning and Functional Breed Selection
Source: Animals (Basel). 2025 Dec 25;16(1):60. doi: 10.3390/ani16010060 (PMC12784992; doi:10.3390/ani16010060)
Supplement: Supplementary file 1 [file animals-16-00060-s001.zip › animals-4054866-supplementary.pdf]

Table S1: Results of the analysis regarding the frequencies of looking at the owner. Significant difference is highlighted with bold.

Fixed factors	df, error	F value	p value
Intercept	1,9	0.059	0.814
Door size	1,9	0.231	0.643
Breed type	1,9	0.129	0.728
Demo	1,9	0.430	0.528
Sex	1,9	5.753	**0.040**
Neutered status	1,9	1.068	0.328
Keeping	1,9	2.094	0.182
Training	5,9	1.227	0.371
CI	72,9	0.578	0.902

Table S2: Results of the analysis regarding the frequencies of looking at the experimenter. Significant difference is highlighted with bold.

Fixed factors	df, error	F value	p value
Intercept	1,9	1.018	0.339
Door size	1,9	0.950	0.355
Breed type	1,9	0.150	0.707
Demo	1,9	0.968	0.351
Sex	1,9	0.443	0.522
Neutered status	1,9	1.775	0.215
Keeping	1,9	0.835	0.385
Training	5,9	0.630	0.683
CI	72,9	1.518	0.257
Trials	5,45	3.373	**0.011**

Table S3: Results of the analysis regarding the frequencies of looking at the door. Significant difference is highlighted with bold.

Fixed factors	df, error	F value	p value
Intercept	1,9	32.625	<0.001
Door size	1,9	2.103	0.181
Breed type	1,9	2.018	0.189
Demo	1,9	0.146	0.711
Sex	1,9	1.238	0.295
Neutered status	1,9	0.089	0.772
Keeping	1,9	3.738	0.085
Training	5,9	1.317	0.338
CI	72,9	1.030	0.529
Trials	5,45	4.139	**0.004**

Table S4: Results of the analysis regarding the frequencies of trying to go through the door. Significant difference is highlighted with bold.

Fixed factors	df, error	F value	p value
Intercept	1,9	9.788	0.012

Door size	1,9	0.038	0.850
Breed type	1,9	0.103	0.756
Demo	1,9	0.414	0.536
Sex	1,9	2.322	0.162
Neutered status	1,9	0.108	0.750
Keeping	1,9	1.310	0.282
Training	5,9	0.286	0.909
CI	72,9	0.955	0.588

Table S5: data of subjects participated in the experiment. L-cont: large door, control; l-demo: large door, detour demonstration; s-cont: small door, control; s-demo: small door, detour demonstration. Age is given in years.

Name	Breed type	Test group	Breed	Height (cm)	Cephalic index	Age	Sex
Füge	cooperative	l-cont	Australian Shepherd	51	52.6	2.5	female
Brandon	cooperative	l-cont	Border Collie	52	49.9	NA	female
Theo	cooperative	l-cont	Border Collie	56	49.4	4	male
Life	cooperative	l-cont	Cocker Spaniel	38	48.31	7	female
Nico	cooperative	l-cont	Cocker Spaniel	41	48.31	2	male
Lilly	cooperative	l-cont	Cocker Spaniel	37	48.31	2	female
Magnum	cooperative	l-cont	German Shepherd Dog	64	54.6	10	male
Bukfenc	cooperative	l-cont	German Shepherd Dog	65	50.1	3.5	female
Mázli	cooperative	l-cont	Golden Retriever	60	55.51	NA	female
Samu	cooperative	l-cont	Golden Retriever	62	50.3	5	male
Flash	cooperative	l-cont	Golden Retriever	62	47.5	4.5	male
Uwel	cooperative	l-cont	Labrador Retriever	57	54.85	2	male
Zoja	cooperative	l-cont	Labrador Retriever	52	58.4	2	female
Panka	cooperative	l-cont	Puli	38	53.7	9	female
Momo	cooperative	l-cont	Shetland Sheepdog	35	49.2	6	female
Pite	cooperative	l-demo	Australian Shepherd	56	57.6	3	male
Bori	cooperative	l-demo	Border Collie	43	53.4	3	female
Mafla	cooperative	l-demo	Boxer	67	74.8	2	male
Karna	cooperative	l-demo	Cocker Spaniel	40	48.31	4.5	male
Lenore	cooperative	l-demo	Cocker Spaniel	30	48.31	7	female
Díva	cooperative	l-demo	Cocker Spaniel	40	48.31	7	female
Kon	cooperative	l-demo	Rough Collie	55	47.1	9	male
Wasabi	cooperative	l-demo	German Shepherd Dog	70	45.1	8	female
Rosie	cooperative	l-demo	German Shepherd Dog	65	52.1	5	female
Lia	cooperative	l-demo	Golden Retriever	56	55.51	3	female
Cody	cooperative	l-demo	Golden Retriever	58	49.9	3	male
Dante	cooperative	l-demo	Labrador Retriever	57	54.85	2	male
Bogi	cooperative	l-demo	Lagotto Romagnolo	45	64.9	4	female
Jet	cooperative	l-demo	Miniature American Shepherd	43	57.1	7	male
Ködmön	cooperative	l-demo	Mudi	43	59.7	10	male
Tökmag	cooperative	l-demo	Mudi	52	59.7	1	male
Mici	cooperative	l-demo	Puli	38	58.7	9	female
Quinn	cooperative	l-demo	Shetland Sheepdog	37	50.5	3	male
Neji	cooperative	s-cont	Australian Shepherd	58	51.2	3	male
Skylar	cooperative	s-cont	Border Collie	48	56.05	1.5	male

Oli	cooperative	s-cont	Briard	69	NA	NA	male
Spam	cooperative	s-cont	Briard	68	45.5	2	male
Jázmin	cooperative	s-cont	Cocker Spaniel	38	48.31	4.5	female
Hope	cooperative	s-cont	Cocker Spaniel	40	48.31	5	female
Lady	cooperative	s-cont	Cocker Spaniel	38	48.31	2	female
Grogu	cooperative	s-cont	German Shepherd Dog	1,5	50.26	1.5	male
Fancy	cooperative	s-cont	Golden Retriever	58	55.51	10	female
Bella	cooperative	s-cont	Irish Setter	62	47.4	5.5	female
Samu	cooperative	s-cont	Labrador Retriever	58	54.2	6	male
Amarena	cooperative	s-cont	Labrador Retriever	53	51.2	2	female
Peti	cooperative	s-cont	Labrador Retriever	63	52.7	12	male
Phoebe	cooperative	s-cont	Lagotto Romagnolo	55	54.8	3	female
Reiko	cooperative	s-cont	Pastore della Lessinia e del Lagorai	62	NA	3	male
Buga	cooperative	s-cont	Poodle	37	55.7	8	male
Hádész	cooperative	s-cont	Rottweiler	64	68.8	6	male
Rudi	independent	l-cont	Airedale Terrier	70	47.1	3	male
Bogi	independent	l-cont	Akita	63	57.0	6	female
Filip	independent	l-cont	Beagle	39	49.2	4.5	male
Vilma	independent	l-cont	Bedlington Terrier	42	51.9	2.5	female
Hippi	independent	l-cont	Bernese Mountain Dog	53	59.5	3	female
Maya	independent	l-cont	Foxterrier	38	45.3	1.5	female
Süti	independent	l-cont	Foxterrier	39	50	NA	NA
Tammie	independent	l-cont	Hungarian Greyhound	66	46.3	3	female
Bodza	independent	l-cont	Miniature Pinscher	33	50.5	1	female
Hamu	independent	l-cont	Miniature Schnauzer	34	60.5	8	female
Kodak	independent	l-cont	Samoyed	54	61.8	1	male
Luna	independent	l-cont	Siberian Husky	64	53.6	4	female
Bodza	independent	l-cont	Transylvanian Hound	65	49.4	6	female
Totó	independent	l-cont	Welsh Terrier	45	58.6	11	male
Akshan	independent	l-cont	Whippet	59	49.2	2.5	male
Kiwi	independent	l-demo	Airedale Terrier	53	44.4	1.5	female
Jade	independent	l-demo	Airedale Terrier	59	51.9	9	male
Afi	independent	l-demo	American Staffordshire Terrier	48	57.9	8	female
Bodza	independent	l-demo	American Staffordshire Terrier	55	61.1	4	female
Sherlock	independent	l-demo	Basset Hound	45	48.7	2	male
Luna	independent	l-demo	Bullterrier	45	51.7	11	female
Maszat	independent	l-demo	German Spitz Mittel	41	66.1	1.5	female
Vatta	independent	l-demo	Hungarian Greyhound	66	42.4	6	female
Jolly	independent	l-demo	Irish Terrier	50	51.8	6	female
Pipacs	independent	l-demo	Irish Terrier	45	42.1	10	female
Csinszka	independent	l-demo	Jack Russel Terrier	31	58.4	3	female
Dexter	independent	l-demo	Miniature Schnauzer	41	50.6	2	male
Iláz	independent	l-demo	Siberian Husky	58	61.4	6	male
Aldo	independent	l-demo	Whippet	42	47.5	2	male
Pixel	independent	l-demo	Whippet	51	45.1	1.5	female
Tangó	independent	s-cont	Basset Hound	37	47.9	1.5	male
Luca	independent	s-cont	Beagle	38	55.6	3.5	female
Due	independent	s-cont	Bullmastiff	71	72.9	6	male

Saca	independent	s-cont	Foxterrier	36	54.9	2.5	female
Dior (Azure Angel)	independent	s-cont	Great Dane	84	48.4	1	male
Levedi	independent	s-cont	Hungarian Greyhound	76	47.3	2	male
Pagi	independent	s-cont	Hungarian Greyhound	66	41.3	1	female
Parázs	independent	s-cont	Irish Terrier	49	43.4	4	male
Jackie	independent	s-cont	Jack Russel Terrier	32	51.6	4	male
Lierde (de Alba de los Danzantes)	independent	s-cont	Pyrenean Mountain Dog	75	47.7	2	female
Dino (Thorwysh du Néouvielle)	independent	s-cont	Pyrenean Mountain Dog	76	64.8	1.5	male
Mimóza	independent	s-cont	Samoyed	58	57.2	4	female
Kira	independent	s-cont	Siberian Husky	58	55.9	5	female
Ikon	independent	s-cont	Transylvanian Hound	61	50.6	7	male
Zara	independent	s-cont	Whippet	52	48.6	3.5	female
Jávor	independent	s-cont	Whippet	50	47.9	8	male
Luigi	cooperative	s-demo	Cocker Spaniel	41	47.3	3	male
Sophie	cooperative	s-demo	Border Collie	48	57.4	3.5	female
Manka	cooperative	s-demo	Collie	48	44.5	5	female
Frutti	cooperative	s-demo	Cardigan Welsh Corgi	33	58.4	3.5	female
Alma	cooperative	s-demo	Mudi	38	57.2	3	female
Zorka	cooperative	s-demo	Groenendael	59	47.9	5	female
Millie	cooperative	s-demo	Collie	51	44.1	13	female
Oreo	cooperative	s-demo	Border Collie	53	52.9	4	male
Griff	cooperative	s-demo	German Shepherd Dog	64	51.5	3	male
Dió	cooperative	s-demo	Labrador Retriever	54	56.1	6	female
Brúnó	cooperative	s-demo	Mudi	49	47.1	1.5	male
Mangó	cooperative	s-demo	Weimaraner	64	46.3	3.5	female
Domino	cooperative	s-demo	Bracco Italiano	58	46.3	1.5	male
Ezra	cooperative	s-demo	Poodle	62	NA	1	male
Sudár	cooperative	s-demo	Mudi	42	54	3	female
Timo	cooperative	s-demo	Golden Retriever	58	49.8	3	male
Lalika	cooperative	s-demo	German Shepherd Dog	67	44.6	8	female
Byron	independent	s-demo	American Staffordshire Terrier	54	67.37	6	male
Luna	independent	s-demo	Dalmatian	59	48.7	2.5	female
Polly	independent	s-demo	Airedale Terrier	55	53	4.5	female
Milka	independent	s-demo	Whippet	58	46.3	4	male
Kiwi	independent	s-demo	Bedlington Terrier	39	49.6	2	female
Thor	independent	s-demo	Hovawart	62	51.7	4	male
Hina	independent	s-demo	Shiba Inu	38	61.2	1.5	female
Robin	independent	s-demo	Siberian Husky	58	54.4	4	female
Houdini	independent	s-demo	Welsh Terrier	41	50.9	7	male
Angel	independent	s-demo	Welsh Terrier	42	49	12	female
Áfonya	independent	s-demo	Italian Greyhound	38	51.6	2.5	female
Deadpool	independent	s-demo	Catahoula	62	44.4	3	male
Luna	independent	s-demo	Italian Greyhound	33	50.5	8	female
Zsiga	independent	s-demo	Whippet	62	47.1	3	male
Faust	independent	s-demo	Italian Greyhound	40	50.9	5	male
